# Functional and Taxonomic Diversity of Anaerobes in Supraglacial Microbial Communities

**DOI:** 10.1128/spectrum.01004-22

**Published:** 2023-03-20

**Authors:** Francesca Pittino, Krzysztof Zawierucha, Ewa Poniecka, Jakub Buda, Asia Rosatelli, Simone Zordan, Roberto S. Azzoni, Guglielmina Diolaiuti, Roberto Ambrosini, Andrea Franzetti

**Affiliations:** a Department of Earth and Environmental Sciences (DISAT)–University of Milano-Bicocca, Milano, Italy; b Biodiversity and Conservation Biology, Swiss Federal Research Institute WSL, Birmensdorf, Switzerland; c Department of Animal Taxonomy and Ecology, Faculty of Biology, Adam Mickiewicz University, Poznań, Poland; d Faculty of Forestry and Wood Sciences, Czech University of Life Sciences, Prague, Czech Republic; e Department of Environmental Microbiology and Biotechnology, Faculty of Biology, University of Warsaw, Warsaw, Poland; f Department of Earth Science “Ardito Desio,” University of Milan, Milano, Italy; g Department of Environmental Science and Policy (ESP), University of Milan, Milano, Italy; University of Texas at San Antonio

**Keywords:** cryoconite, metatranscriptomics, extremophiles

## Abstract

Cryoconite holes are small ponds present on the surface of most glaciers filled with meltwater and sediment at the bottom. Although they are characterized by extreme conditions, they host bacterial communities with high taxonomic and functional biodiversity. Despite that evidence for a potential niche for anaerobic microorganisms and anaerobic processes has recently emerged, the composition of the microbial communities of the cryoconite reported so far has not shown the relevant presence of anaerobic taxa. We hypothesize that this is due to the lower growth yield of anaerobes compared to aerobic microorganisms. In this work, we aim at evaluating whether the anaerobic bacterial community represents a relevant fraction of the biodiversity of the cryoconite and at describing its structure and functions. We collected sediment samples from cryoconite holes on the Forni Glacier (Italy) and sequenced both 16S rRNA amplicon genes and 16S rRNA amplicon transcripts at different times of the day along a clear summer day. Results showed that a relevant fraction of taxa has been detected only by 16S rRNA transcripts and was undetectable in 16S rRNA gene amplicons. Furthermore, in the transcript approach, anaerobic taxa were overrepresented compared with DNA sequencing. The metatranscriptomics approach was used also to investigate the expression of the main metabolic functions. Results showed the occurrence of syntrophic and commensalism relationships among fermentative bacteria, hydrogenothrophs, and consumers of fermentation end products, which have never been reported so far in cryoconite.

**IMPORTANCE** Recent evidence disclosed the presence of a potential niche for anaerobic microorganisms and anaerobic processes in supraglacial sediments (cryoconite), but a detailed description of the structure and functions of the anaerobic population is still lacking. This work used rRNA and mRNA sequencing and demonstrated that anaerobes are very active in these environments and represent a relevant albeit neglected part of the ecosystem functions in these environments.

## INTRODUCTION

Cryoconite holes are small water-filled ponds with sediment at the bottom that are present on the surface of most glaciers during the ablation season. Although they are characterized by extreme conditions such as low nutrient content, permanently low temperatures, and high solar irradiance, they host microbial communities with high taxonomic and functional biodiversity (1). It has been supposed that such high diversity could be due to the high versatility of some of the most abundant populations, which, in turn, is due to their adaptive response to the dynamic-changing conditions of the supraglacial habitats (2, 3). In a previous investigation, we demonstrated that these supraglacial communities exhibit high functional biodiversity since they exploit organic matter both as an energy and carbon source, and use both oxygenic and anoxygenic photosynthesis with pure autotrophic and mixotrophic lifestyles (4). Cryoconite was considered an oxygen-rich environment until recent times when pieces of evidence for a potential niche for anaerobic microorganisms and anaerobic processes emerged (5). Indeed, as a habitat rich in organic matter on the ice surface, it offers a suitable environment for anaerobes, also thanks to the presence of small biogenic granules formed by microbial activity. According to measurements in the field (5), the anoxic zone in the cryoconite holes can represent two-thirds of the sediment depth, which is corroborated by laboratory measurements (6). A similar anoxic layer was found also in the cryoconite of the Forni glacier (our unpublished data), a large valley glacier in the Italian Alps. However, the composition of the microbial communities of the Forni cryoconite reported so far did not show a relevant presence of anaerobic taxa on this glacier. This may be due to the lower growth yield of anaerobes compared to aerobic microorganisms (7), which may result in a high bias toward dominant taxa when using PCR-based library preparation for widely used DNA metabarcoding, which can magnify the initial disproportion of DNA sequences. If this hypothesis were true, the biodiversity of the cryoconite may be potentially higher than currently estimated with the DNA metabarcoding approach. Consequently, also our understanding of the metabolic interactions and, more generally, of the ecology of cryoconite holes would be severely biased. Since glaciers are one of the fastest disappearing cryospheric ecosystems (8), we need to improve tools and methods for describing their biodiversity and ecology with no bias.

In this paper, we aim at testing the hypothesis that the anaerobic bacterial populations represent a relevant fraction of the biodiversity of the cryoconite and are metabolically active. We hypothesized that the analysis of 16S ribosomal RNA transcripts (rRNA) might better detect active but slow-growing bacterial populations compared to the most common analysis of 16S ribosomal DNA amplicon genes (rDNA), thus allowing a more accurate description of the anaerobic microbial populations and their ecosystem functions. For our study, we collected cryoconite samples from four cryoconite holes on the Forni Glacier (Italy) and sequenced both 16S rDNA and 16S rRNA. In addition, to investigate a possible diurnal turnover in the active bacterial community, we collected samples from each hole at different times of the day along a clear summer day. These cryoconite holes were stable during the sampling day without changes of shape or removal of sediments by meltwater. Finally, on a subset of samples, we also used shotgun metatranscriptomics sequencing to investigate the expression of the main metabolic functions along the day. Cryoconite holes are optimal model ecosystems for these studies because of their simplicity such as stable temperatures (0.1 to 0.5°C) and a short and truncated trophic web and are an example of shallow reservoirs maintaining permanent water and allowing sunlight penetration to the bottom (3).

## RESULTS

### Alpha diversity.

**FIG 1:**
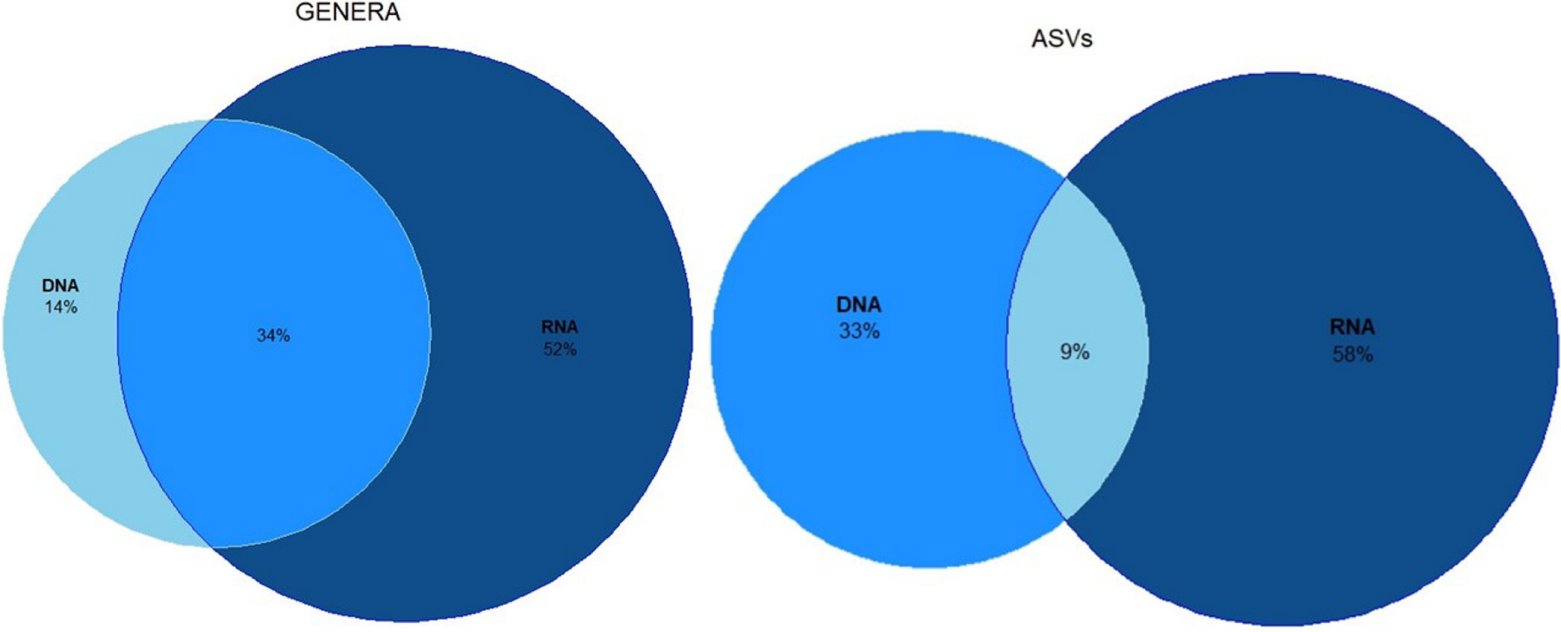
Venn diagram showing the distribution of bacterial genera and ASVs in DNA and RNA samples. The overlapping areas contain the shared genera between the two types of samples. The subgroups with the higher percentages have lighter shades of blue, while those with lower ones have darker shades.

The Venn diagrams in Fig. 1 report the exclusive and shared amplicon sequences variants (ASVs) and the classified genera between rDNA and rRNA data sets, including the samples for which we have both rDNA and rRNA sequencing.

Results of this analysis showed that both at the genus and the ASVs levels, the inclusion of rRNA in the analyses led to an increase of the detected taxa compared with rDNA only. The highest percentage of genera was present in rRNA samples (52%), the smallest fraction in rDNA samples (14%), and an intermediate fraction was shared (34%) between rRNA and rDNA samples. At the ASV level, we obtained similar results. The rRNA samples contained the highest percentage of ASVs (58%), while the lowest was shared between rDNA and rRNA (9%), while an intermediate percentage was found in rDNA only (33%). Generalized linear models (GLMs) showed that both Shannon and Gini indexes varied significantly with the genetic material (rDNA or rRNA) (*F*_1,14_ ≥ 12.70, false discovery rate *P* [*P*_FDR_] ≤ 0.009; Fig. S1 in the supplemental material), revealing higher values of the Shannon index and lower values of the Gini index in rRNA than in rDNA samples. However, the same pattern was not observed for the number of ASVs (*F*_1,14_ = 3.631, *P*_FDR_ = 0.142). No variation was observed in the alpha-diversity indexes according to sampling time (*F*_3,14_ ≤ 0.406, *P*_FDR_ ≥ 0.751) and cryoconite hole (*F*_3,14_ ≤ 3.713, *P*_FDR_ ≥ 0.205) in any analysis.

### Beta diversity.

**FIG 2:**
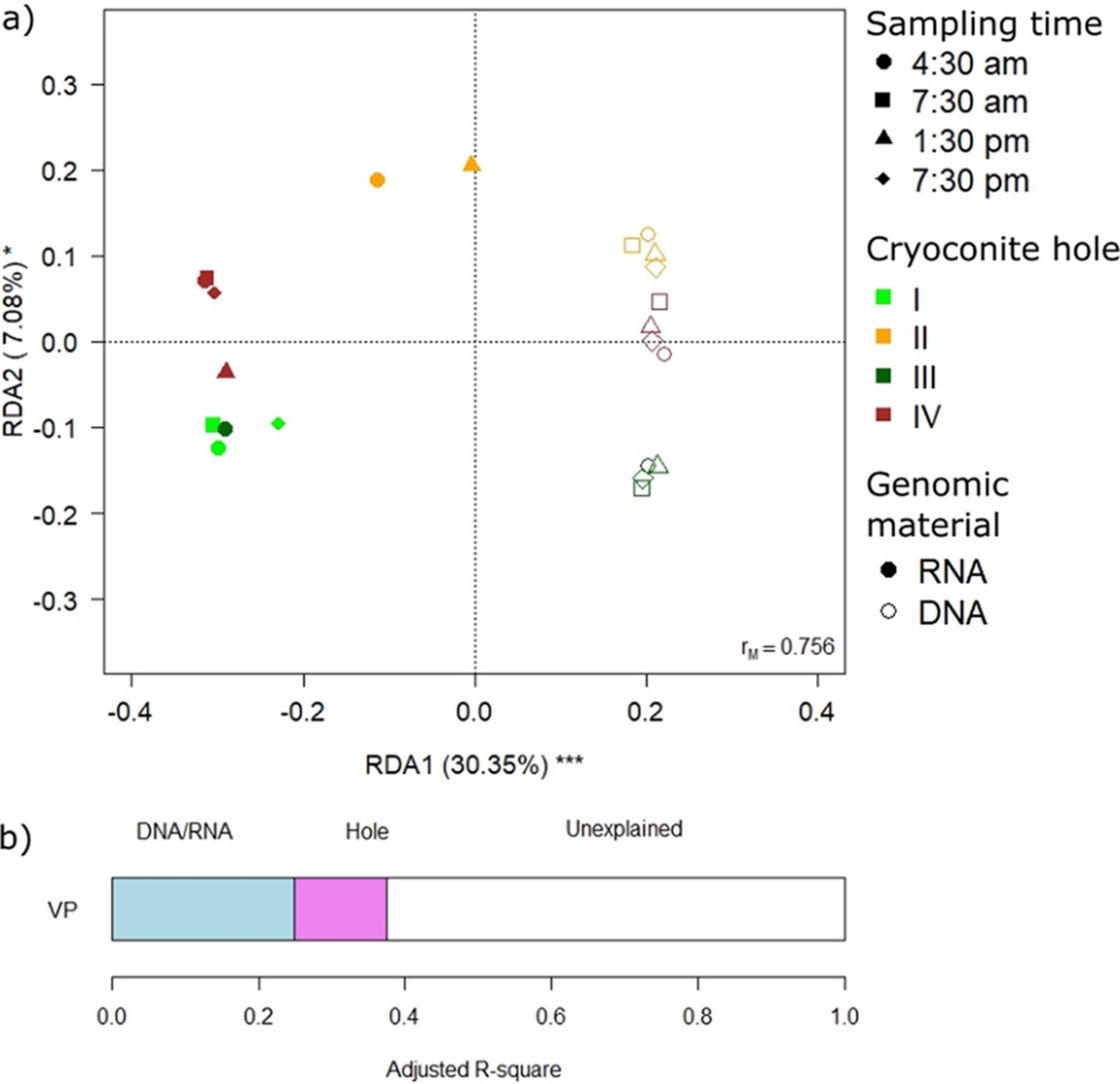
(a) Biplot from RDA on Hellinger-transformed bacterial ASV abundance on time, cryoconite hole identity, and type of sample (DNA/RNA). Each point represents one sample. Samples collected in different holes are indicated by different colors, while different polygons indicate the type of samples (squares, RNA; circles, DNA) The percentage of variance explained by each axis and its significance (***, *P* < 0.001) is reported. *r*_M_ is the Mantel correlation coefficient between the Hellinger distance between samples and the Euclidean distance between the corresponding symbols in the graph. Values close to one indicate that the graph correctly represents the distance between samples. (b) Results from the variation partitioning (VP) showing the amount of variance explained by the independent effects of the predictors entered in the RDA. There was no combined effect of the two significant variables.

The redundancy analysis (RDA) performed on amplicon data, including the type of genetic material, the cryoconite hole, and time as predictors, confirmed that the structure of the observed communities differed between rDNA and rRNA (Fig. 2). In addition, community structures differed between cryoconite holes but not between sampling times (Table 1). The *post hoc* tests showed that all the holes were different from one another (*F*_5,15_ ≥ 1.909, *P*_FDR_ ≤ 0.029) except for holes I and III, which did not differ (*F*_1,5_ = 1.96, *P*_FDR_ = 0.08). Variation partitioning analysis showed that the type of genetic material explained more variance than the hole identity (Fig. 2).

**TABLE 1:** RDA of variation of Hellinger-transformed bacterial ASV abundance of all the samples according to the type of samples (DNA/RNA), time, and sampled hole^*a*^

Variable	Df	Variance	*F*	*P*
DNA/RNA	1	0.136	7.316	0.001
Time	3	0.0493	0.887	0.590
Hole	3	0.119	2.136	0.006
Residual	14	0.259		

a *F*_7,14_ = 2.795, *P* = 0.001; adjusted *R*² = 0.358.

### Metabolic traits of bacterial populations.

**FIG 3:**
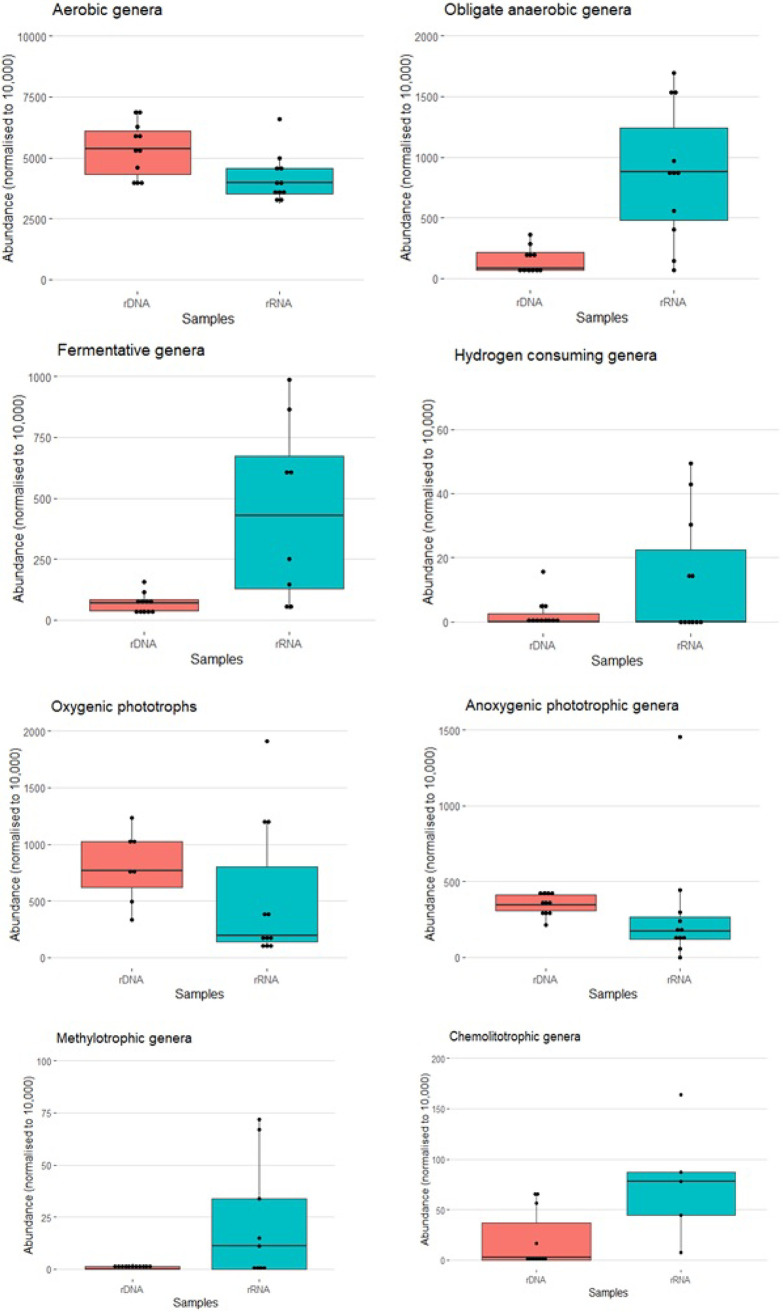
Boxplot of the relative abundance of bacterial genera (except for Cyanobacteria that are reported at class level) for the investigated metabolic traits. Genera whose abundance was <0.1% are not reported. *, Significant difference between the mean values (*P* < 0.05).

In the amplicon data sets, the main energy metabolic traits were investigated, including all the classified genera with a relative abundance >0.1%. Figure 3 shows the sum of the relative abundances of aerobic, obligated anaerobic, H_2_-metabolizing, methylotroph, lithotrophic, anoxygenic phototrophic, oxygenic phototrophic, and fermentative bacteria according to the genetic material (rDNA or rRNA). Paired *t* tests on samples with both rRNA and rDNA data showed that aerobic genera were more abundant in rDNA than in rRNA (*t*_7_ = 5.018, *P*_FDR_ = 0.012), while obligate anaerobic genera were more abundant in rRNA than in rDNA (*t*_7_ = −4.173, *P*_FDR_ = 0.029). All the other metabolisms investigated did not differ significantly between rDNA and rRNA (*P*_FDR_ ≥ 0.052). The taxonomic classification of the metabolic groups is reported in Fig. S2. The results of quantitative PCR (qPCR) quantification of 16S rRNA and *nar*G (Table S1) indicated that the copy numbers of the 16S RNA gene were in the orders of magnitude of 10^9^ to 10^10^ per gram of cryoconite and their transcripts varied between 1.3 × 10^6^ and 8.9 × 10^6^ copy number per gram due to the lower extraction yield of RNA compared with DNA. *nar*G gene copies resulted to be 1 order of magnitude lower than their respective 16S rRNA genes; conversely, *nar*G transcript copies exceeded by 3 orders of magnitude the copy numbers of the 16S RNA transcripts.

### Shotgun metatranscriptomics.

Shotgun metatranscriptomics was carried out on the 4 samples collected from hole II. Table 2 reports the marker gene transcripts whose coverage (mean number per base of reads mapping the genes) was used to infer the expression of each metabolism and their normalized coverages. We report the coverage of all transcribed genes with the annotated Kegg Orthology number and the taxonomic attribution (Supplemental Material S2). We focused on the main energy and carbon metabolisms and the results confirmed that phototrophic metabolisms were actively expressed throughout the day but not at night. Autotropic carbon fixation was also active throughout the day and both aerobic and anaerobic respiration were active.

**TABLE 2:** Transcript coverages of the marker genes

				Coverage			
Metabolism	Gene	Short name	KO orthology	4:30 am	7:30 am	1:30 pm	7:30 pm
Oxygenic photosynthesis	Photosystem II P680 reaction center D2 protein	*psb*D	K02706	2.1	317.4	83.8	163.5
Aerobic anoxygenic photosynthesis	Photosynthetic reaction center L and M subunits	*puf*M	K08929	3.2	32.4	0.0	69.6
CO_2_ fixation, Calvin-Benson cycle	Ribulose bisphosphate carboxylase small and large chain (RubisCO)	*rbc*L	K01602, K01601	25.5	96.8	84.2	50.0
Chitin assimilation	Chitinase	*chi*A	K01183	68.7	6.5	0.0	35.1
Hydrolysis *O*- and *S*-glycosyl compounds	Endo-1,4-beta-xylanase	*xyn*A	K01181	ND	ND	ND	ND
Carbon monoxide oxidation	Carbon-monoxide dehydrogenase medium and large subunits	*cox*ML	K03519, K03520	ND^*a*^	ND	ND	ND
Aerobic respiration	Cytochrome *c* oxidase subunit I	*cox*A	K02274	908.8	283.4	677.6	735.1
Dissimilatory nitrate reduction (denitrification/nitrification)	Nitrate reductase alpha subunit	*nar*G	K00370	748.0	145.8	568.0	454.8
Dissimilatory nitrate reduction (denitrification)	Nitrite reductase (nitric oxide-forming) [EC:1.7.2.1]	*nir*K	K00368	831.3	129.3	778.2	408.5
Dissimilatory nitrate reduction (denitrification)	Nitric oxide reductase subunit B [EC:1.7.2.5]	*norB*	K04561	815.5	105.8	666.3	409.1
Ammonia oxidation (nitrification)	Ammonia monooxygenase A and B subunits	*amo*A	K10944	0.0	1.0	0.1	0.1
Sulfur oxidation	Sulfur-oxidizing protein	*sox*A	K17222	0.0	13.3	0.0	40.3
Hydrogen oxidation	Hydrogenase large subunit	*hya*B	K06281	19.0	0.0	26.0	12.5
Nitrogen fixation	Nitrogenase iron protein	*nif*H	K02588	0.2	0.0	0.3	2.4

a ND, not detected.

## DISCUSSION

Glacial ecosystems gained special attention in the last few years due to their unique physical characteristics, unique biodiversity, and rapid global disappearing (9). The biodiversity of cryoconite has been discussed in the literature (10–12) but, only in the last years, have the tools of molecular biology allowed the investigation of the majority of glacial organisms that are invisible with classical microscopy (13). However, our knowledge of the taxonomic groups and functional diversity of glaciers is still limited and this prevents our understanding of how these ecosystems function or what organisms may disappear along with melting ice.

In this study, we used both DNA- and RNA-based 16S rRNA gene sequencing to investigate the biodiversity of cryoconite holes and to accurately describe the composition of the anaerobic bacterial communities.

### Biodiversity assessment based on rDNA and rRNA approach.

The RDA, which included the type of nucleic acid and the time and the sampling site as predictors (Fig. 2), confirmed that the structure of the communities assessed based on rDNA was different from that assessed based on rRNA. So far, few studies have investigated the differences between genomic and transcriptomic data in supraglacial environments (see, e.g., references 14, 15), whereas such differences have been already reported for other environments (16–20). It is normally expected that the whole community is described by rDNA sequencing, whereas rRNA sequencing provides information about the active fraction of the community. However, if undetected populations with rDNA sequencing express a high number of rRNA transcripts, they could be detectable only in the metatranscriptomes. As shown by the Venn diagrams (Fig. 1), only 9% of the ASVs are shared between rDNA and rRNA, 69% appear exclusively in rRNA sequences and only 22% are exclusive of rDNA sequences. This result might suggest that it is likely that the active part of the bacterial communities in cryoconite holes is only a small fraction of the whole community present in these microhabitats and remains undetected if we use rDNA sequencing only probably because it is masked by the more abundant DNA of the predominant but less active part. Consistently, the Venn diagram, including all the classified genera (Fig. 3) showed that most genera (52%) were classified from rRNA analyses only. So far, most of the studies have characterized the bacterial communities of cryoconite holes at the order or phylum level, and the most abundant phyla reported were *Proteobacteria* (alpha and beta), *Actinobacteria*, *Chloroflexi*, *Acidobacteria*, *Cyanobacteria*, and *Bacteroidetes* (21–23), while the most typical orders were *Sphingobacteriales*, *Pseudomonadales*, *Rhodospirillales*, *Burkholderiales*, and *Clostridiales* (24). All these taxa were found in both DNA and RNA analyses, and this confirms the role of the above-mentioned taxa as the representative ones of cryoconite hole bacterial communities. The only exception was the phylum *Chloroflexi*, which was detected exclusively in RNA, likely because it was not one of the most abundant phyla in our samples (<1%), and their DNA was probably masked by that of the more abundant but inactive taxa. This result is consistent with those already reported by Anesio et al. (25), who hypothesized that only a minor fraction of the cryoconite bacterial community is active. This hypothesis is further supported by the results reported in Fig. 3, which show that anaerobic taxa are more abundant in rRNA rather than in rDNA. Since it is now well known that cryoconite holes are anoxic environments, we can state the aerobic community that is predominant in the rDNA data are likely inactive.

### Diversity and functions of the anaerobic community.

In the diversity detected using rRNA analysis, anaerobic taxa were overrepresented compared with rDNA analysis (Fig. 3). This seems to confirm our hypothesis of the occurrence of an active anaerobic community in cryoconite holes and suggests that the studies of cryoconite might have underestimated the actual anaerobic diversity present in these disappearing environments. Stibal et al. (14) also found differences between the rDNA and rRNA data in the composition of communities in marginal and interior sites of the Greenland Ice Sheet. However, in this work, the composition at the phylum level did not show the presence of anaerobic taxa, and they were not found among the dominant taxa in rRNA data. Furthermore, the estimated richness based on the rRNA approach was generally lower than that estimated by rDNA.

Microsensor data registered *in situ* in the cryoconite of Forni glacier (unpublished) showed that the oxic part of the cryoconite on Forni Glacier measured in the field is restricted to the upper layer (ca. 400 μm), which represents a minor fraction of the cryoconite mass, consistently with the results published by Poniecka et al. (5). On the other hand, the interior of cryoconite granules serves as a habitat for anaerobic communities (15). On Forni, such structures are 0.2 to 1.3 mm in diameter and might host anaerobes even the oxygenic cryoconite-water interface. In addition, cryoconite is inhabited by small invertebrates like tardigrades and rotifers. On Forni Glacier, the densities of tardigrades in cryoconite holes reach 172 specimens per mL of sediment (26). The microbiome of these animals is represented by anoxic bacteria that can enrich the pool of anaerobes found by the rRNA analyses in the cryoconite hole ecosystem (27). We put forward that the upper and oxic layer of the cryoconite hosts most of the bacterial biomass whereas the lower and anoxic layer, together with the inner part of cryoconite granules, contain less abundant but extremely active populations. Only a few studies by Segawa and coworkers (15, 28) addressed the microbial communities in the redox-stratified layers of cryoconite. In particular, Segawa et al. (15) analyzed the structure and function of the bacterial communities in redox-stratified cryoconite granules, using both rDNA- and rRNA-based 16S rRNA sequencing. The results showed that also in that case the taxonomic compositions differed considerably between the two approaches. In the rDNA-based analysis, Cyanobacteria were more abundant in the surface layer than in the inner core and they were the dominant phylum in the rRNA-based analyses. Interestingly, despite among the major bacterial genera found in rRNA-based analyses no obligate anaerobic taxa were recorded, the authors found an increase of denitrification-related genes in the inner core of granules compared to the surface layer. Our results are also consistent with those reported by Zdanowski et al. (29), who showed that anaerobic bacteria dominated cryoconite communities after an 8-week incubation under strictly anoxic conditions. The authors of that paper inferred that cryoconite microbes might contribute to the taxonomic, eco-physiologic, and genetic diversity of the subglacial habitat, which is widely recognized to host active anaerobic microbial populations under glaciers and ice sheets worldwide. Our results are also in agreement with those reported by Poniecka et al. (3) who isolated and characterized anaerobic bacteria in Polar cryoconite.

*Clostridium*, *Propionibacterium*, *Syntrophobacter*, and candidatus *Cloacamonas* were the most abundant obligate anaerobic genera in our rRNA samples (Fig. S2 in Supplemental Material S1). *Clostridium* members are obligate anaerobic bacteria that have been already reported in Forni cryoconite at high abundance at the beginning of the melting season (30). *Propionibacterium* members are strictly anaerobic bacteria able to produce propionic acid during fermentation. This genus has been mainly described as human associated, but its presence in cold environments has been already documented as part of the Actinobacterial communities of Arctic marine sediments (31). Members of *Syntrophobacter* are described as strictly anaerobic chemoorganotrophs, which grow by syntrophic metabolism and sulfate reduction. Interestingly, owing to their ability to oxidize propionate in the presence of H_2_-consuming organisms, they could establish commensalism relationships with *Propionibacterium* and syntrophic relationships with the hydrogenotrophic populations, retrieved in rRNA data (Fig. S1 - Supplemental Material S1) and, likely, also with methanogenic archaea, which are not included in the amplicon data. However, the transcripts of some genes of the methanogenesis pathway have been annotated in the shotgun metatranscriptome, namely, formylmethanofuran dehydrogenase subunit (*fmd*A), formylmethanofuran-tetrahydromethanopterin *N*-formyltransferase (*ftr*), methenyltetrahydromethanopterin cyclohydrolase (*mch*), and heterodisulfide reductase (*hdr*B2) (Supplemental Material S2), which suggest that methanogenic archaea were present in our samples, but were not detected because we did not use primers specific for archaea.

Interestingly, the hydrogenotrophic bacteria classified in rRNA data displayed different energy metabolisms: denitrification (*Paracoccus*), acetogenesis (*Acetobacterium*, *Acetanaerobium*), and sulfate reduction (*Desulfovibrio*). The presence of methanogenic archaea can be inferred also by the detection in the rRNA data of methylotrophs such as *Methylobacterium*, *Methylotenera*, and *Methylocaldum* (Fig. S1 - Supplemental Material S1), which can feed on the methane produced by methanogenic archaea. Candidatus *Cloacamonas* members are uncultured bacteria mainly found in anaerobic digesters but also described in anoxic soils and sediments (32). A recent reconstruction of the complete genome of candidatus *Cloacamonas acidaminovorans* revealed that these bacteria can grow by the fermentation of amino acids, sugars, and carboxylic acids. Particularly, their genome contains all the genes involved in the obligate syntrophic pathway for the oxidation of propionate into acetate and carbon dioxide (32). Therefore, this taxon can also contribute to the consumption of propionate produced by *Propionibacterium*. It is worth mentioning that among the taxa overrepresented in the rRNA data, besides anaerobic ones, other low-growth-yield taxa are present, like methylotrophs and chemolithotrophs (Fig. S1 - Supplemental Material S1).

The shotgun metatranscriptomics allowed describing the metabolic function associated with the active bacterial community of the cryoconite during the daytime. The results showed that different carbon and energy metabolisms are expressed throughout the day (Table 2). Aerobic respiration and denitrification were the dominant terminal electron-accepting processes, and both oxygenic and anoxygenic phototrophy genes were actively transcribed at different levels along the day. According to the taxonomic affiliation of *cox*A and *nir*K, *Actinobacteria*, and *Proteobacteria* were the most active taxa involved in respiration with slightly different abundances along the day (Supplemental Material S2).

mRNA sequencing confirmed that anaerobic respiration actively occurred in cryoconite holes, in agreement with rRNA amplicon data. However, the detected functional gene transcripts were restricted to denitrification. Indeed, the complete denitrification pathway genes (*nar*G, *nir*K, and *nor*B) were transcribed as already reported in the cryoconite of a Chinese glacier (15, 28). However, neither iron reduction (rusticyanin) nor dissimilatory sulfite reductase gene (*dsr*A) transcripts were detected (Supplemental Material S2), in disagreement with the amplicon rRNA data where sulfate reducers have been detected. This inconsistency could be explained by the higher coverage of amplicon sequencing compared with shotgun sequencing.

As expected, Cyanobacteria and algae were active oxygenic phototrophs. Aerobic anoxygenic phototrophs (APPs) resulted affiliated with alpha and betaproteobacteria (Supplemental Material S2) and are known to be photoheterotrophic, thus using the organic matter as a carbon source (the organic matter on Forni varied from 5% to 50% and differed between habitats and seasons; reference 26) and complementing their energy demand with light (33). A relevant expression of the CO_2_ fixation genes (*rblc*L) was observed at all sampling times. Indeed, *Cyanobacteria* and algae were the most active CO_2_ fixators. However, according to the taxonomic affiliation of *rblc*L, *Proteobacteria* were the most active primary producers at night and at 1:30 pm, thus suggesting that chemolithoautotrophy could be active. Despite ammonia oxidizers (*Nitrosomonas* and *Nitrosospira*) have been detected among the chemolithotrophic genera in the rRNA amplicons, the expression of *amo*A (ammonia monooxygenase A subunit) was negligible at any time. Conversely, the transcription of *nar*G, which codes for nitrate reductase alpha subunit, was found. This protein is involved in the oxidation of nitrite to nitrate, thus its presence is in agreement with the detection of *Nitrospira* in the rRNA amplicons. Carbon monoxide oxidation to CO_2_ was previously hypothesized as a possible lithotrophic metabolism in cryoconite due to the high abundance of carbon-monoxide dehydrogenase (*cox*ML) (4). However, the transcripts of this gene were not retrieved in this study. Among the different key genes for lithotrophic sulfur oxidation (*apr*A and *dsr*A), only *sox*A was transcribed (Supplemental Material S2). The presence and activity of sulfur-oxidizing bacteria were supported by the presence of *Sulfuricella* among the classified genera in both rDNA and rRNA amplicons. However, *sox* genes are also harbored by anoxygenic phototrophs (34).

As previously mentioned, hydrogen seems to be actively utilized by chemolithotrophs. Indeed, different H_2_-consuming genera have been retrieved both under oxic and anoxic conditions. Consistently, shotgun metatranscriptomics data showed that hydrogenase (*hya*B) genes were actively transcribed (Table 2). This transcription was particularly high when CO_2_ fixation was due to nonphototrophs (4.30 a.m. and 1.30 pm). This might indicate that hydrogen oxidation might provide alternative reducing power to photosynthesis. This report of shotgun metatranscriptomics in cryoconite, therefore, shows that different microbial taxa contribute to the same ecosystem functions (e.g., carbon fixation and respiration) along the day and primary productivity was supported by both photo- and chemosynthesis. This functional redundancy likely enhances ecosystem stability in such extreme environments.

In summary, our study showed that only the use of integrative molecular approaches may facilitate the study of microbial biodiversity and functions in the cryosphere. For example, rare microbial taxa play an important role in cryoconite environments (35); however, often they are overlooked by using only one approach. Indeed, rRNA analysis revealed active groups that were undetectable by rDNA alone, which implies that our knowledge about the functionality of cryoconite microbial communities is far from complete. Moreover, the activity of primary producers during the 24-h period suggests that a complete understanding of carbon budget and primary productivity in cryoconite ecosystems requires analyses at different spatial and temporal frames. Here, we provided a framework for the recognition of anaerobic microbial communities and the activity of microbial groups in cryoconite holes along the day, demonstrating not only that the cryoconite environment on an alpine glacier is codominated by anaerobic communities but also that it is functionally more complex than previously hypothesized.

## MATERIALS AND METHODS

### Sampling and DNA/RNA extraction.

Cryoconite was collected from four shallow cryoconite holes (I, II, III, and IV) at four different times (4:30 am, 7:30 am, 1:30 pm, and 7:30 pm) on 24 July 2018. The cryoconite holes were located on the ablation tongue of the Forni Glacier (Italy, 46.39868° N, 10.58664° E) at 2,670 m above sea level. To preserve the RNA content, cryoconite was immediately mixed with an RNA preservative solution (1:5 volume ratio) prepared with 4 mL of EDTA 0.5 M, 2.5 mL of 1 M Na_3_C_6_H_5_O_7_, and 70 g of (NH_4_)_2_SO_4_, DEPC-H_2_O for a final volume of 100 mL (36) and stored at −80°C within 48 h.

Total DNA was extracted from 0.5 g of each cryoconite sample with the FastDNA Spin for Soil kit (MP Biomedicals, Solon, OH, USA) according to the manufacturer’s instructions. A detailed description is presented in reference 10. Total RNA was extracted from 1 g of cryoconite with the RNeasy PowerSoil Total RNA kit (Qiagen, Hilden, Germany) according to the manufacturer’s instructions. After the extraction, residual DNA was removed with the RQ1 RNase-free DNase (Promega) from 6 μL of extracted RNA.

### Sequencing of bacterial 16S rRNA genes and 16S rRNA transcripts.

The extracted RNA was retrotranscribed with the RevertAid First Strand cDNA Synthesis kit (Thermo Scientific, Waltham, MA, USA) using random primers. V5 to V6 region of the 16S rRNA gene was amplified and sequenced using both extracted DNA and cDNA as the templates, as previously reported (10). Sequences were then demultiplexed according to the indexes and clustered in ASVs with DADA2 (37). Samples with a total abundance of ASVs lower than 2,400 were discarded. The classification of ASVs was carried out using the RDP classifier (confidence 50%) (38). The table of abundance at the genus level was normalized with a total abundance equal to 10,000 including only classified ASVs. Physiological traits were identified at the genus level for genera with at least 80 ASVs in all the samples (on average, at least 10 ASVs per time of sampling in both rDNA and rRNA samples). The following traits were assigned based on the genus description paper (retrieved at https://lpsn.dsmz.de/): obligate anaerobic, H_2_-metabolizing, methylotroph, lithotrophic, anoxygenic phototrophic, oxygenic phototrophic, and fermentative bacteria.

### Shotgun metatranscriptomics.

The total DNA-free RNA from each sample was treated with the MICROBExpress Bacterial mRNA Enrichment kit (Ambion) and 8 μL of the depleted RNA were retrotranscribed with the RevertAid First Strand cDNA Synthesis kit (Thermo Scientific, Waltham, MA, USA) using random primers. Shotgun sequencing was applied to cDNA by HiSeq Illumina (Illumina, Inc., San Diego, CA, USA) using a 150-bp × 2 paired-end protocol on one lane. The paired-end reads were quality-trimmed (minimum length, 80 bp; minimum average quality score, 30) using Sickle (https://github.com/najoshi/sickle).

Filtered reads were coassembled using IDBA-UD (39) that iterated the value of k_mer_ from 40 to 99 (with a step of 5). Predicted genes were inferred from contigs with Prodigal (40). KEGG orthology (KO) numbers were assigned to the predicted proteins using the online tool GhostKOALA (41). The lowest common ancestor algorithm was applied to infer the taxonomic affiliation of predicted genes using MEGAN with default parameters (42). Hierarchical taxonomic data were visualized with Krona (43). The average per-base coverage of the predicted genes was calculated using filtered reads with bowtie2 (44, 45) and bedtools (46). To normalize the different sequencing depths across samples, the sum of gene coverages was normalized to 600,000 per sample.

### qPCR analyses.

Quantification of the number of copies of 16S rRNA and molecular markers for denitrification (narG) was performed through qPCR on PCRmax ECO 48 real-time PCR system (PCR max, Stone, UK) on both DNA and cDNA. The analyses were carried out on three samples. The primer set used to target the rRNA 16S 466-bp fragment (331 to 797 according to Escherichia coli position) included 331 forward (5′-TCCTACGGGAGGCAGCAGT-3′) and 797 reverse (5′-GGACTACCAGGGTATCTAATCCTGTT-3′). narG encodes the subunit alpha of respiratory nitrate reductase and was used as a marker gene for nitrate reduction, while forward primer *nar*G 1960F (5′-TAYGTSGGGCAGGARAAACTG-3′) and reverse primer *nar*G 2050R (5′-CGTAGAAGAAGCTGGTGCTGTT-3′) were used to amplify a 109-bp fragment of the aforementioned gene.

The quantification for each marker of interest was performed on both DNA extract and cDNA obtained through the retro transcription of the RNA extract. Each reaction was carried out in triplicate in a total volume of 10 μL using the Luna Universal qPCR Master Mix (NEB, MA, USA) with 0.3 μM forward and reverse primer final concentration. Each assay included a control without the template. To confirm the specific amplification of the target gene, melting curves were obtained by heating amplicons.

The amplification cycle for the 16S rRNA gene consisted of an initial DNA denaturation at 95°C for 4 min, followed by 40 cycles of denaturation at 95°C for 15 sec, primers annealing at 60°C for 30 sec, and extension at 72°C for 15 sec. Fluorescence was recorded at the end of each elongation step. A final cycle (95°C for 15 sec, 55°C per 15 sec, and 95°C for 15 sec) was performed to obtain dissociation curves.

The same steps conducted at different temperatures were used to quantify the genes targeted for microbial anaerobic metabolism. The amplification cycle consisted of initial DNA denaturation (95°C for 4 min), followed by 40 cycles of denaturation (95°C for 15 sec), primers annealing (53°C for 30 sec) and extension (72°C for 15 sec). Fluorescence was recorded at the end of each elongation step. Dissociation curves were produced as follows: 95°C for 15 sec, 55°C per 15 sec, and 95°C for 15 sec.

To quantify the PCR products, standard curves based on threshold values were obtained through the amplification of 10-fold dilutions series of standard DNA obtained through the generation of a plasmid containing the target regions using each primer set.

### Statistical analyses.

Singletons (ASVs present in one sample only) were removed from the ASVs table. Statistical analyses were performed on a nonrarefied data set with R 3.5.1 (47) with the following packages: VEGAN, BIODIVERSITYR, MULTTEST, EULERR, GGPLOT2, and MULTCOMP. The number of ASVs present in a sample was used as a measure of alpha diversity. Venn diagrams were produced including only those samples for which we obtained enough sequences from both rRNA and rDNA. The samples that were excluded were as follows: hole I (all four sampling times for which we did not obtain DNA data), hole II, and hole III (samples collected at 7:30 a.m. and 7:30 p.m.). The Shannon (48) and Gini (49) indexes were used as further measures of alpha diversity. They were calculated on a data set rarefied to 2,400 sequences per sample, i.e., slightly less than the minimum number of sequences in the sample with the lowest number of sequences. GLMs were performed to investigate changes in alpha diversity indexes according to sampling time, sampled cryoconite hole, and type of genetic material (RNA or DNA). RDA and variation partitioning were performed to obtain the variation of the community structure (beta diversity) between rDNA and rRNA and between sampling times (included as a four-level factor) and different cryoconite holes. These analyses were based on the Hellinger distance, which decreases the importance of coabsences when comparing the ASVs composition of different samples (50, 51). Pairwise differences between holes were tested with *post hoc* tests (Tukey method), correcting *P* values for multiple statistical tests according to the false discovery rate (FDR) procedure (52).

### Data availability.

Sequence data were submitted to NCBI SRA database, BioProject number 816448 (https://www.ncbi.nlm.nih.gov/sra/PRJNA816448).

## References

[1] Cook J, Edwards A, Takeuchi N, Irvine-Fynn T. 2015. Cryoconite: the dark biological secret of the cryosphere. Prog Phys Geogr 40:66–111. doi:10.1177/0309133315616574.

[2] Darcy JL, Lynch RC, King AJ, Robeson MS, Schmidt SK. 2011. Global distribution of polaromonas phylotypes–evidence for a highly successful dispersal capacity. PLoS One 6:e23742. doi:10.1371/journal.pone.0023742. 21897856 PMC3163589

[3] Poniecka EA. Physiological capabilities of cryoconite hole microorganisms. 2020. Front Microbiol 11:1–14. doi:10.3389/fmicb.2020.00001. 32849402 PMC7412143

[4] Franzetti A, Tagliaferri I, Gandolfi I, Bestetti G, Minora U, Mayer C, Azzoni RS, Diolaiuti G, Smiraglia C, Ambrosini R. 2016. Light-dependent microbial metabolisms drive carbon fluxes on glacier surfaces. ISME J 10:2984–2988. doi:10.1038/ismej.2016.72. 27128995 PMC5148193

[5] Poniecka EA, Bagshaw EA, Tranter M, Sass H, Williamson CJ, Anesio AM. 2018. Rapid development of anoxic niches in supraglacial ecosystems. Arctic Antarct Alp Res 50:1. doi:10.1080/15230430.2017.1420859.

[6] Buda J, Poniecka EA, Rozwalak P, Ambrosini R, Bagshaw EA, Franzetti A, Klimaszyk P, Nawrot A, Pietryka M, Richter D, Zawierucha K. 2022. Is oxygenation related to the decomposition of organic matter in cryoconite holes? Ecosystems 25:1510–1521. doi:10.1007/s10021-021-00729-2.

[7] Capdeville B, Nguyen KM. 1990. Kinetics and modelling of aerobic and anerobic film growth. Water Sci Technol 22:149–170. doi:10.2166/wst.1990.0077.

[8] Fountain AG, Campbell JL, Schuur EA, Stammerjohn SE, Williams ME, Ducklow HW. 2012. The disappearing cryosphere: impacts and ecosystem responses to rapid cryosphere loss. Bioscience 62:405–415. doi:10.1525/bio.2012.62.4.11.

[9] Stibal M, Šabacká M, Žárský J. 2012. Biological processes on glacier and ice sheet surfaces. Nat Geosci 5:771–774. doi:10.1038/ngeo1611.

[10] Pittino F, Maglio M, Gandolfi I, Azzoni RS, Diolaiuti G, Ambrosini R, Franzetti A. 2018. Bacterial communities of cryoconite holes of a temperate alpine glacier show both seasonal trends and year-to-year variability. Ann Glaciol 59:1–9. doi:10.1017/aog.2018.16.

[11] Segawa T, Yonezawa T, Edwards A, Akiyoshi A, Tanaka S, Uetake J, Irvine-Fynn T, Fukui K, Li Z, Takeuchi N. 2017. Biogeography of cryoconite forming cyanobacteria on polar and Asian glaciers. J Biogeogr 44:2849–2861. doi:10.1111/jbi.13089.

[12] Margesin R, Zacke G, Schinner F. 2002. Characterization of heterotrophic microorganisms in alpine glacier cryoconite. Arctic Antarct Alp Res 34:88–93. doi:10.1080/15230430.2002.12003472.

[13] Hodson A, Anesio AM, Tranter M, Fountain A, Osborn M, Priscu J, Laybourn-Parry J, Sattler B. 2008. Glacial ecosystems. Ecol Monogr 78:41–67. doi:10.1890/07-0187.1.

[14] Stibal M, Schostag M, Cameron KA, Hansen LH, Chandler DM, Wadham JL, Jacobsen CS. 2015. Different bulk and active bacterial communities in cryoconite from the margin and interior of the Greenland ice sheet. Environ Microbiol Rep 7:293–300. doi:10.1111/1758-2229.12246. 25405749

[15] Segawa T, Takeuchi N, Mori H, Rathnayake RMLD, Li Z, Akiyoshi A, et al. 2020. Redox stratification within cryoconite granules influences the nitrogen cycle on glaciers. FEMS Microbiol Ecol 96:199. doi:10.1093/femsec/fiaa199. 32990745

[16] Nogales B, Moore ERB, Llobet-Brossa E, Rossello-Mora R, Amann R, Timmis KN. 2001. Combined use of 16S ribosomal DNA and 16S rRNA To study the bacterial community of polychlorinated biphenyl-polluted soil. Appl Environ Microbiol 67:1874–1884. doi:10.1128/AEM.67.4.1874-1884.2001. 11282645 PMC92809

[17] Eichler S, Christen R, Höltje C, Westphal P, Bötel J, Brettar I, Mehling A, Höfle MG. 2006. Composition and dynamics of bacterial communities of a drinking water supply system as assessed by RNA- and DNA-based 16S rRNA gene fingerprinting. Appl Environ Microbiol 72:1858–1872. doi:10.1128/AEM.72.3.1858-1872.2006. 16517632 PMC1393179

[18] Gentile G, Giuliano L, D'Auria G, Smedile F, Azzaro M, De Domenico M, Yakimov MM. 2006. Study of bacterial communities in Antarctic coastal waters by a combination of 16S rRNA and 16S rDNA sequencing. Environ Microbiol 8:2150–2161. doi:10.1111/j.1462-2920.2006.01097.x. 17107556

[19] Razzauti M, Galan M, Bernard M, Maman S, Klopp C, Charbonnel N, et al. 2015. A comparison between transcriptome sequencing and 16S metagenomics for detection of bacterial pathogens in wildlife. PLoS Negl Trop Dis 9:e0003929. doi:10.1371/journal.pntd.0003929. 26284930 PMC4540314

[20] De Vrieze J, Pinto AJ, Sloan WT, Ijaz UZ. 2018. The active microbial community more accurately reflects the anaerobic digestion process: 16S rRNA (gene) sequencing as a predictive tool. Microbiome 6:1–13. doi:10.1186/s40168-017-0383-2. 29609653 PMC5879801

[21] Boetius A, Anesio AM, Deming JW, Mikucki JA, Rapp JZ. 2015. Microbial ecology of the cryosphere: sea ice and glacial habitats. Nat Rev Microbiol 13:677–690. doi:10.1038/nrmicro3522. 26344407

[22] Christner BC, Kvitko BH, Reeve JN. 2003. Molecular identification of bacteria and Eukarya inhabiting an Antarctic cryoconite hole. Extremophiles 7:177–183. doi:10.1007/s00792-002-0309-0. 12768448

[23] Ambrosini R, Musitelli F, Navarra F, Tagliaferri I, Gandolfi I, Bestetti G, Mayer C, Minora U, Azzoni RS, Diolaiuti G, Smiraglia C, Franzetti A. 2017. Diversity and assembling processes of bacterial communities in cryoconite holes of a karakoram glacier. Microb Ecol 73:827–837. doi:10.1007/s00248-016-0914-6. 27999874

[24] Franzetti A, Navarra F, Tagliaferri I, Gandolfi I, Bestetti G, Minora U, Azzoni RS, Diolaiuti G, Smiraglia C, Ambrosini R. 2017. Potential sources of bacteria colonizing the cryoconite of an Alpine glacier. PLoS One 12:e0174786. doi:10.1371/journal.pone.0174786. 28358872 PMC5373619

[25] Anesio AM, Sattler B, Foreman C, Telling J, Hodson A, Tranter M, Psenner R. 2010. Carbon fluxes through bacterial communities on glacier surfaces. Ann Glaciol 51:32–40. doi:10.3189/172756411795932092.

[26] Zawierucha K, Buda J, Azzoni RS, Niśkiewicz M, Franzetti A, Ambrosini R. 2019. Water bears dominated cryoconite hole ecosystems: densities, habitat preferences and physiological adaptations of Tardigrada on an alpine glacier. Aquat Ecol 53:543–556. doi:10.1007/s10452-019-09707-2.

[27] Zawierucha K, Trzebny A, Buda J, Bagshaw E, Franzetti A, Dabert M, et al. 2022. Trophic and symbiotic links between obligate-glacier water bears (Tardigrada) and cryoconite microorganisms. PLoS One 17:e0262039. doi:10.1371/journal.pone.0262039. 35020747 PMC8754347

[28] Segawa T, Ishii S, Ohte N, Akiyoshi A, Yamada A, Maruyama F, Li Z, Hongoh Y, Takeuchi N. 2014. The nitrogen cycle in cryoconites: naturally occurring nitrification-denitrification granules on a glacier. Environ Microbiol 16:3250–3262. doi:10.1111/1462-2920.12543. 24946985

[29] Zdanowski MK, Bogdanowicz A, Gawor J, Gromadka R, Wolicka D, Grzesiak J. 2016. Enrichment of cryoconite hole anaerobes: implications for the subglacial microbiome. Microb Ecol 73:532–538. doi:10.1007/s00248-016-0886-6. 27822618 PMC5348551

[30] Franzetti A, Navarra F, Tagliaferri I, Gandolfi I, Bestetti G, Minora U, Azzoni RS, Diolaiuti G, Smiraglia C, Ambrosini R. 2017. Temporal variability of bacterial communities in cryoconite on an alpine glacier. Environ Microbiol Rep 9:71–78. doi:10.1111/1758-2229.12499. 27897429

[31] Zhang G, Cao T, Ying J, Yang Y, Ma L. 2014. Diversity and novelty of actinobacteria in Arctic marine sediments. Antonie Van Leeuwenhoek 105:743–754. doi:10.1007/s10482-014-0130-7. 24519808

[32] Pelletier E, Kreimeyer A, Bocs S, Rouy Z, Gyapay G, Chouari R, Rivière D, Ganesan A, Daegelen P, Sghir A, Cohen GN, Médigue C, Weissenbach J, Le Paslier D. 2008. “Candidatus Cloacamonas Acidaminovorans”: genome sequence reconstruction provides a first glimpse of a new bacterial division. J Bacteriol 190:2572. doi:10.1128/JB.01248-07. 18245282 PMC2293186

[33] Koblížek M. 2015. Ecology of aerobic anoxygenic phototrophs in aquatic environments. FEMS Microbiol Rev 39:854–870. doi:10.1093/femsre/fuv032. 26139241

[34] Hell R, Dahl C, Knaff D, Leustek T. 2008. Sulfur metabolism in phototrophic organisms. Springer, New York.

[35] Chen Y, Liu Y, Liu K, Ji M, Li Y. 2022. Snowstorm enhanced the deterministic processes of the microbial community in cryoconite at Laohugou Glacier, Tibetan Plateau. Front Microbiol 12:784273. doi:10.3389/fmicb.2021.784273. 35154026 PMC8829297

[36] Gray MA, Pratte ZA, Kellogg CA. 2013. Comparison of DNA preservation methods for environmental bacterial community samples. FEMS Microbiol Ecol 83:468–477. doi:10.1111/1574-6941.12008. 22974342

[37] Callahan BJ, McMurdie PJ, Rosen MJ, Han AW, Johnson AJA, Holmes SP. 2016. DADA2: high-resolution sample inference from Illumina amplicon data. Nat Methods 13:581–583. doi:10.1038/nmeth.3869. 27214047 PMC4927377

[38] Wang Q, Garrity GM, Tiedje JM, Cole JR. 2007. Naive Bayesian classifier for rapid assignment of rRNA sequences into the new bacterial taxonomy. Appl Environ Microbiol 73:5261–5267. doi:10.1128/AEM.00062-07. 17586664 PMC1950982

[39] Peng Y, Leung HCM, Yiu SM, Chin FYL. 2012. IDBA-UD: a de novo assembler for single-cell and metagenomic sequencing data with highly uneven depth. Bioinformatics 28:1420–1428. doi:10.1093/bioinformatics/bts174. 22495754

[40] Hyatt D, Chen G-L, Locascio PF, Land ML, Larimer FW, Hauser LJ. 2010. Prodigal: prokaryotic gene recognition and translation initiation site identification. BMC Bioinformatics 11:119. doi:10.1186/1471-2105-11-119. 20211023 PMC2848648

[41] Kanehisa M, Goto S. 2000. KEGG : Kyoto Encyclopedia of Genes and Genomes. Nucleic Acids Res 28:27–30. doi:10.1093/nar/28.1.27. 10592173 PMC102409

[42] Huson DH, Mitra S, Ruscheweyh H-J, Weber N, Schuster SC. 2011. Integrative analysis of environmental sequences using MEGAN4. Genome Res 21:1552–1560. doi:10.1101/gr.120618.111. 21690186 PMC3166839

[43] Ondov BD, Bergman NH, Phillippy AM. 2011. Interactive metagenomic visualization in a Web browser. BMC Bioinformatics 12:385. doi:10.1186/1471-2105-12-385. 21961884 PMC3190407

[44] Langmead B, Salzberg SL. 2012. Fast gapped-read alignment with Bowtie 2. Nat Methods 9:357–359. doi:10.1038/nmeth.1923. 22388286 PMC3322381

[45] Li H, Handsaker B, Wysoker A, Fennell T, Ruan J, Homer N, Marth G, Abecasis G, Durbin R, 1000 Genome Project Data Processing Subgroup. 2009. The Sequence Alignment/Map format and SAMtools. Bioinformatics 25:2078–2079. doi:10.1093/bioinformatics/btp352. 19505943 PMC2723002

[46] Quinlan AR, Hall IM. 2010. BEDTools: a flexible suite of utilities for comparing genomic features. Bioinformatics 26:841–842. doi:10.1093/bioinformatics/btq033. 20110278 PMC2832824

[47] R Development Core Team 3.0.1. 2013. A language and environment for statistical computing. R Foundation for Statistical Computing, Vienna, Austria. https://www.R-project.org.

[48] Shannon CE. 1948. A mathematical theory of communication. Bell Syst Tech J 27:379–423. doi:10.1002/j.1538-7305.1948.tb01338.x.

[49] Gini C. 1912. Variabilità e mutabilità: contributo allo studio delle distribuzioni e delle relazioni statische.

[50] Legendre P, Legendre L. 2001. Ecologically meaningful transformations for ordination of species data. Oecologia 129:271–280. doi:10.1007/s004420100716. 28547606

[51] De Caceres M, Legendre P, Moretti M. 2010. Improving indicator species analysis by combining groups of sites. Oikos 119:1674–1684. doi:10.1111/j.1600-0706.2010.18334.x.

[52] Benjamini Y, Yekutieli D. 2001. The control of the false discovery rate in multiple testing under dependency. Ann Stat 29:1165–1188.

