# Supplementary material for: Functional and Taxonomic Diversity of Anaerobes in Supraglacial Microbial Communities
Source: Microbiol Spectr. 2023 Mar 20;11(2):e01004-22. doi: 10.1128/spectrum.01004-22 (PMC10100660; doi:10.1128/spectrum.01004-22)
Supplement: Supplemental file 1 — Fig. S1 and S2 and Table S1. Download spectrum.01004-22-s0001.pdf, PDF file, 0.4 MB [file spectrum.01004-22-s0001.pdf]

SUPLEMENTARY MATERIAL

Functional and taxonomic diversity of anaerobes in supraglacial microbial communities

Francesca Pittino[1], Krzysztof Zawierucha[2], Ewa Poniecka[3], Jakub Buda[2], Asia Rosatelli[1], Simone Zordan[1], Roberto S. Azzoni[4], Guglielmina Diolaiuti[5], Roberto Ambrosini[5], Andrea Franzetti[1]

[1] Dept. of Earth and Environmental Sciences (DISAT) - University of Milano-Bicocca, Milano, ITALY

[2] Department of Animal Taxonomy and Ecology, Faculty of Biology, Adam Mickiewicz University, Poznań, Poland

[3] Department of Environmental Microbiology and Biotechnology, Faculty of Biology, University of Warsaw, Warsaw, Poland

[4.] Department of Earth Science "Ardito Desio", University of Milan, Milano, ITALY

[5.] Department of Environmental Science and Policy (ESP), University of Milan, Milano, ITALY

*Corresponding author:

Andrea Franzetti - Dept. of Earth and Environmental Sciences (DISAT) - University of Milano-Bicocca, Milano, Piazza della Scienza 1, 20126 Milano – ITALY Phone +39 02 64482927 - Email: andrea.franzetti@unimib.it

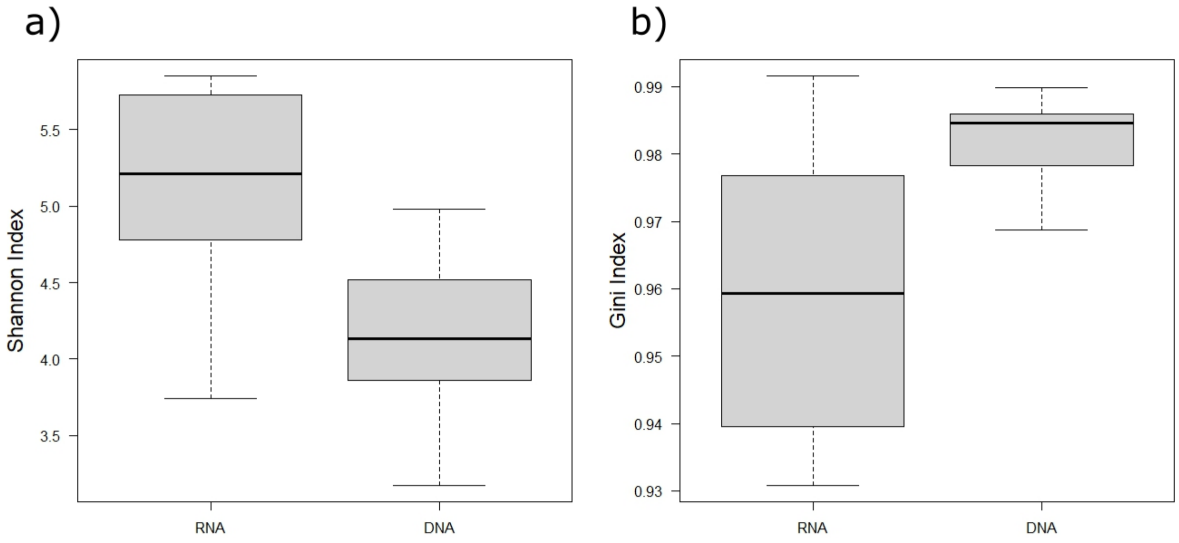

Figure S1 - ***Boxplots of Shannon (a) and Gini (b) indexes of cryoconite holes bacterial communities showing results in different genetic material (RNA and DNA). The thick lines represent the median, boxes upper and lower limits the 25th and the 75th percentiles respectively, whiskers the data that go beyond the 5th and the 75th percentile, dots represent the outliers and different letters indicate significant differences between glaciers at post-hoc tests***

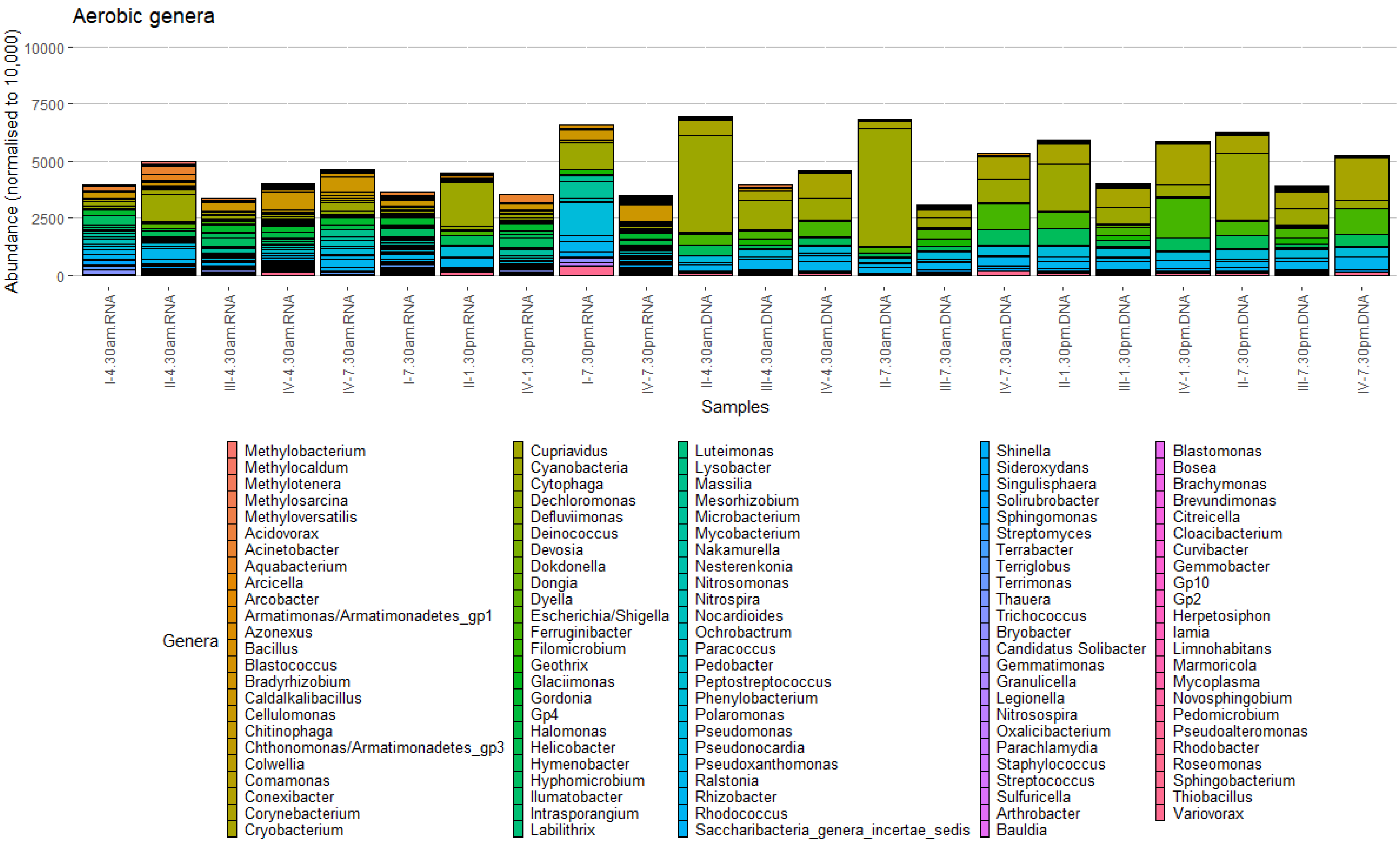
Aerobic genera
Abundance (normalised to 10,000)
10000
7500
5000
2500
0
I-4.30am.RNA
II-4.30am.RNA
III-4.30am.RNA
IV-4.30am.RNA
IV-7.30am.RNA
I-7.30am.RNA
II-1.30pm.RNA
IV-1.30pm.RNA
I-7.30pm.RNA
IV-7.30pm.RNA
II-4.30am.DNA
III-4.30am.DNA
IV-4.30am.DNA
II-7.30am.DNA
III-7.30am.DNA
IV-7.30am.DNA
II-1.30pm.DNA
III-1.30pm.DNA
IV-1.30pm.DNA
II-7.30pm.DNA
III-7.30pm.DNA
IV-7.30pm.DNA
Samples
Genera
Methylobacterium
Methylocaldum
Methylotenera
Methylosarcina
Methyloversatilis
Acidovorax
Acinetobacter
Aquabacterium
Arcicella
Arcobacter
Armatimonas/Armatimonadetes_gp1
Azonexus
Bacillus
Blastococcus
Bradyrhizobium
Caldalkalibacillus
Cellulomonas
Chitinophaga
Chthonomonas/Armatimonadetes_gp3
Colwellia
Comamonas
Conexibacter
Corynebacterium
Cryobacterium
Cupriavidus
Cyanobacteria
Cytophaga
Dechloromonas
Defluviimonas
Deinococcus
Devosia
Dokdonella
Dongia
Dyella
Escherichia/Shigella
Ferruginibacter
Filomicrobium
Geothrix
Glaciimonas
Gordonia
Gp4
Halomonas
Helicobacter
Hymenobacter
Hyphomicrobium
Ilumatobacter
Intrasporangium
Labilithrix
Luteimonas
Lysobacter
Massilia
Mesorhizobium
Microbacterium
Mycobacterium
Nakamurella
Nesterenkonia
Nitrosomonas
Nitrospira
Nocardioides
Ochrobactrum
Paracoccus
Pedobacter
Peptostreptococcus
Phenylobacterium
Polaromonas
Pseudomonas
Pseudonocardia
Pseudoxanthomonas
Ralstonia
Rhizobacter
Rhodococcus
Saccharibacteria_genera_incertae_sedis
Shinella
Sideroxydans
Singulisphaera
Solirubrobacter
Sphingomonas
Streptomyces
Terrabacter
Terriglobus
Terrimonas
Thauera
Trichococcus
Bryobacter
Candidatus Solibacter
Gemmatimonas
Granulicella
Legionella
Nitrosospira
Oxalicibacterium
Parachlamydia
Staphylococcus
Streptococcus
Sulfuricella
Arthrobacter
Bauldia
Blastomonas
Bosea
Brachymonas
Brevundimonas
Citreicella
Cloacibacterium
Curvibacter
Gemmobacter
Gp10
Gp2
Herpetosiphon
Iamia
Limnohabitans
Marmoricola
Mycoplasma
Novosphingobium
Pedomicrobium
Pseudoalteromonas
Rhodobacter
Roseomonas
Sphingobacterium
Thiobacillus
Variovorax

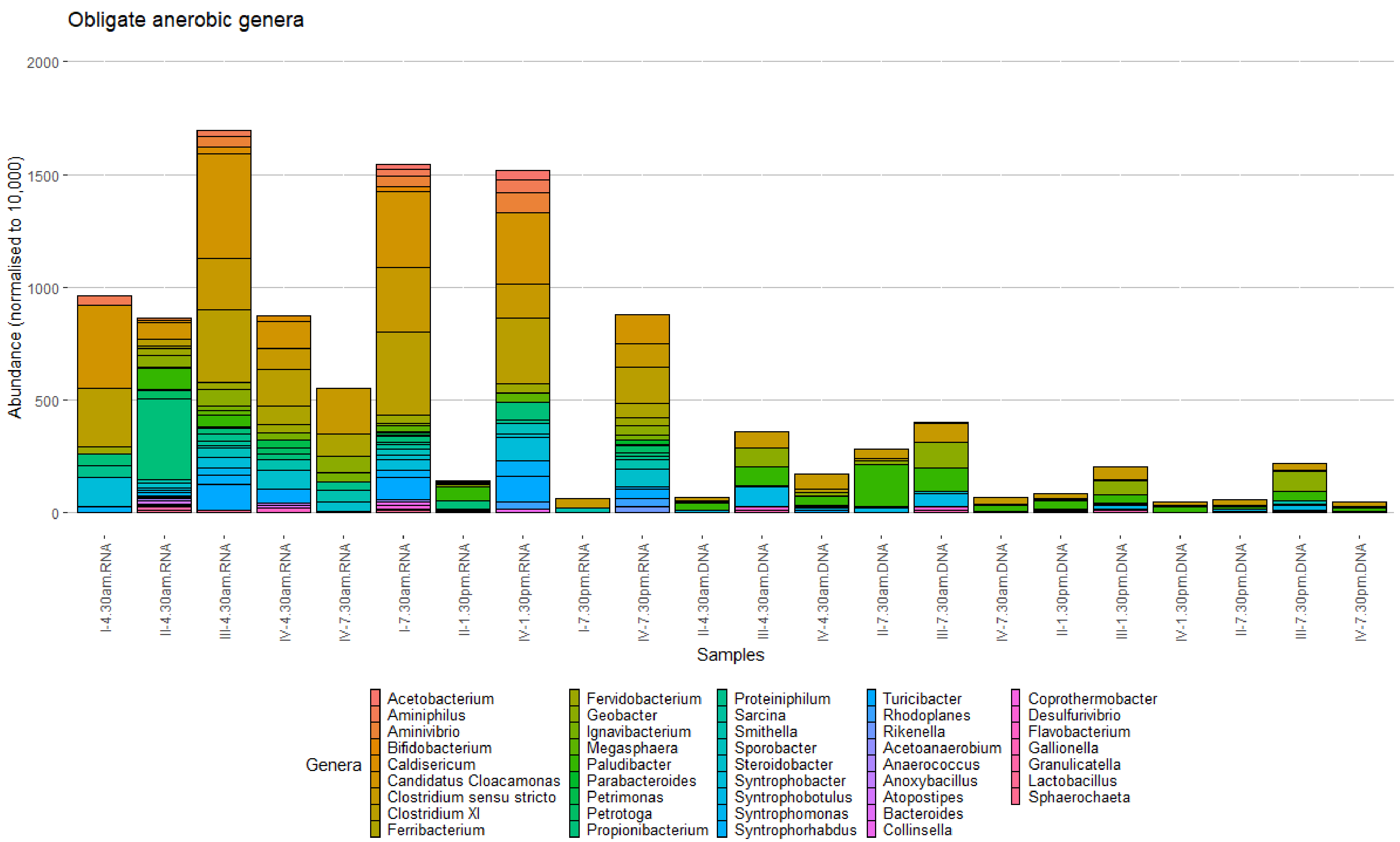
Obligate anerobic genera
Abundance (normalised to 10,000)
2000
1500
1000
500
0
I-4.30am.RNA
II-4.30am.RNA
III-4.30am.RNA
IV-4.30am.RNA
IV-7.30am.RNA
I-7.30am.RNA
II-1.30pm.RNA
IV-1.30pm.RNA
I-7.30pm.RNA
IV-7.30pm.RNA
II-4.30am.DNA
III-4.30am.DNA
IV-4.30am.DNA
II-7.30am.DNA
III-7.30am.DNA
IV-7.30am.DNA
II-1.30pm.DNA
III-1.30pm.DNA
IV-1.30pm.DNA
II-7.30pm.DNA
III-7.30pm.DNA
IV-7.30pm.DNA
Samples
Genera
Acetobacterium
Aminiphilus
Aminivibrio
Bifidobacterium
Caldisericum
Candidatus Cloacamonas
Clostridium sensu stricto
Clostridium XI
Ferribacterium
Fervidobacterium
Geobacter
Ignavibacterium
Megasphaera
Paludibacter
Parabacteroides
Petrimonas
Petrotoga
Propionibacterium
Proteiniphilum
Sarcina
Smithella
Sporobacter
Steroidobacter
Syntrophobacter
Syntrophobotulus
Syntrophomonas
Syntrophorhabdus
Turicibacter
Rhodoplanes
Rikenella
Acetoanaerobium
Anaerococcus
Anoxybacillus
Atopostipes
Bacteroides
Collinsella
Coprothermobacter
Desulfurivibrio
Flavobacterium
Gallionella
Granulicatella
Lactobacillus
Sphaerochaeta

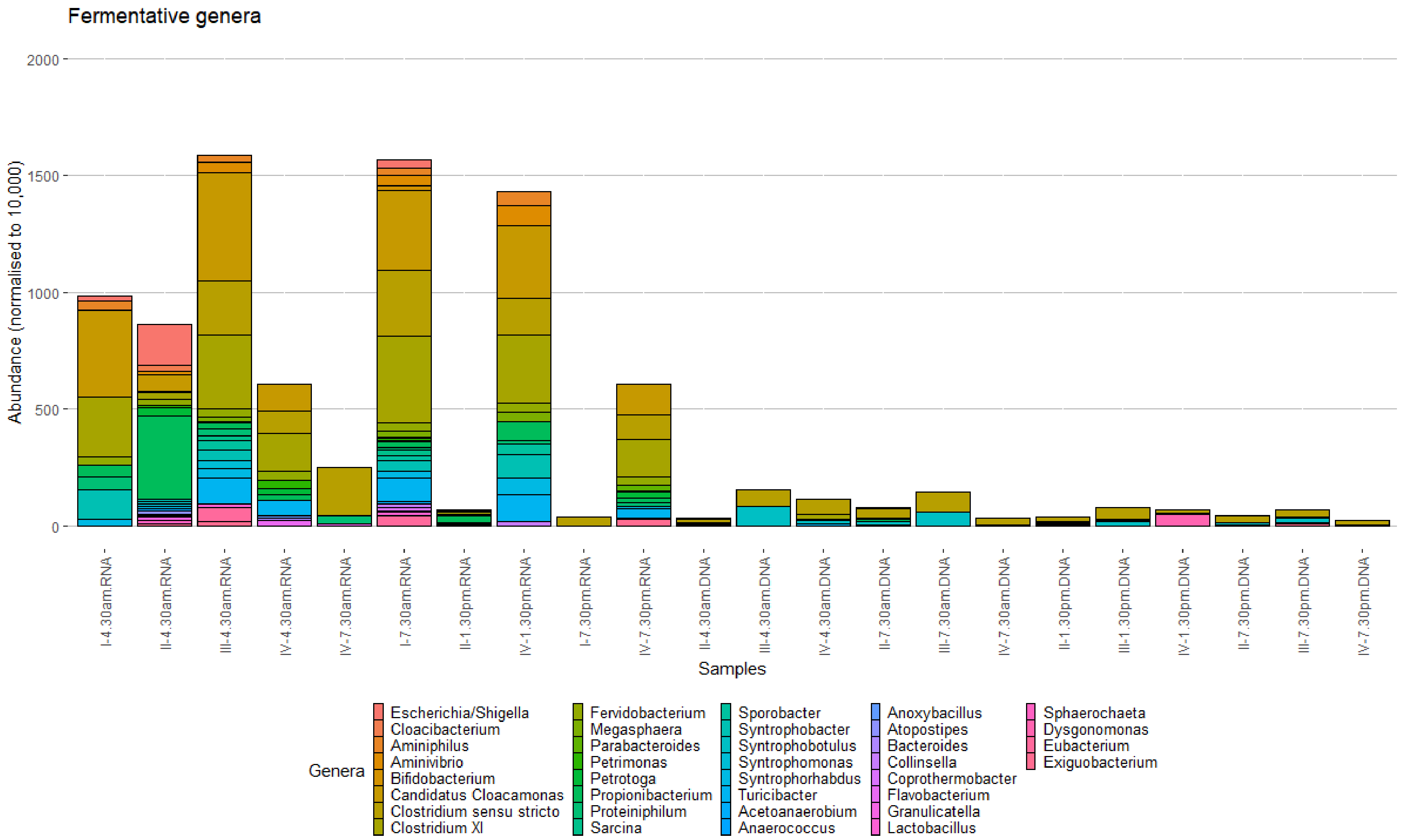
Fermentative genera
Abundance (normalised to 10,000)
2000
1500
1000
500
0
I-4.30am.RNA
II-4.30am.RNA
III-4.30am.RNA
IV-4.30am.RNA
IV-7.30am.RNA
I-7.30am.RNA
II-1.30pm.RNA
IV-1.30pm.RNA
I-7.30pm.RNA
IV-7.30pm.RNA
II-4.30am.DNA
III-4.30am.DNA
IV-4.30am.DNA
II-7.30am.DNA
III-7.30am.DNA
IV-7.30am.DNA
II-1.30pm.DNA
III-1.30pm.DNA
IV-1.30pm.DNA
II-7.30pm.DNA
III-7.30pm.DNA
IV-7.30pm.DNA
Samples
Genera
Escherichia/Shigella
Cloacibacterium
Aminiphilus
Aminivibrio
Bifidobacterium
Candidatus Cloacamonas
Clostridium sensu stricto
Clostridium XI
Fervidobacterium
Megasphaera
Parabacteroides
Petrimonas
Petrotoga
Propionibacterium
Proteiniphilum
Sarcina
Sporobacter
Syntrophobacter
Syntrophobotulus
Syntrophomonas
Syntrophorhabdus
Turicibacter
Acetoanaerobium
Anaerococcus
Anoxybacillus
Atopostipes
Bacteroides
Collinsella
Coprothermobacter
Flavobacterium
Granulicatella
Lactobacillus
Sphaerochaeta
Dysgonomonas
Eubacterium
Exiguobacterium

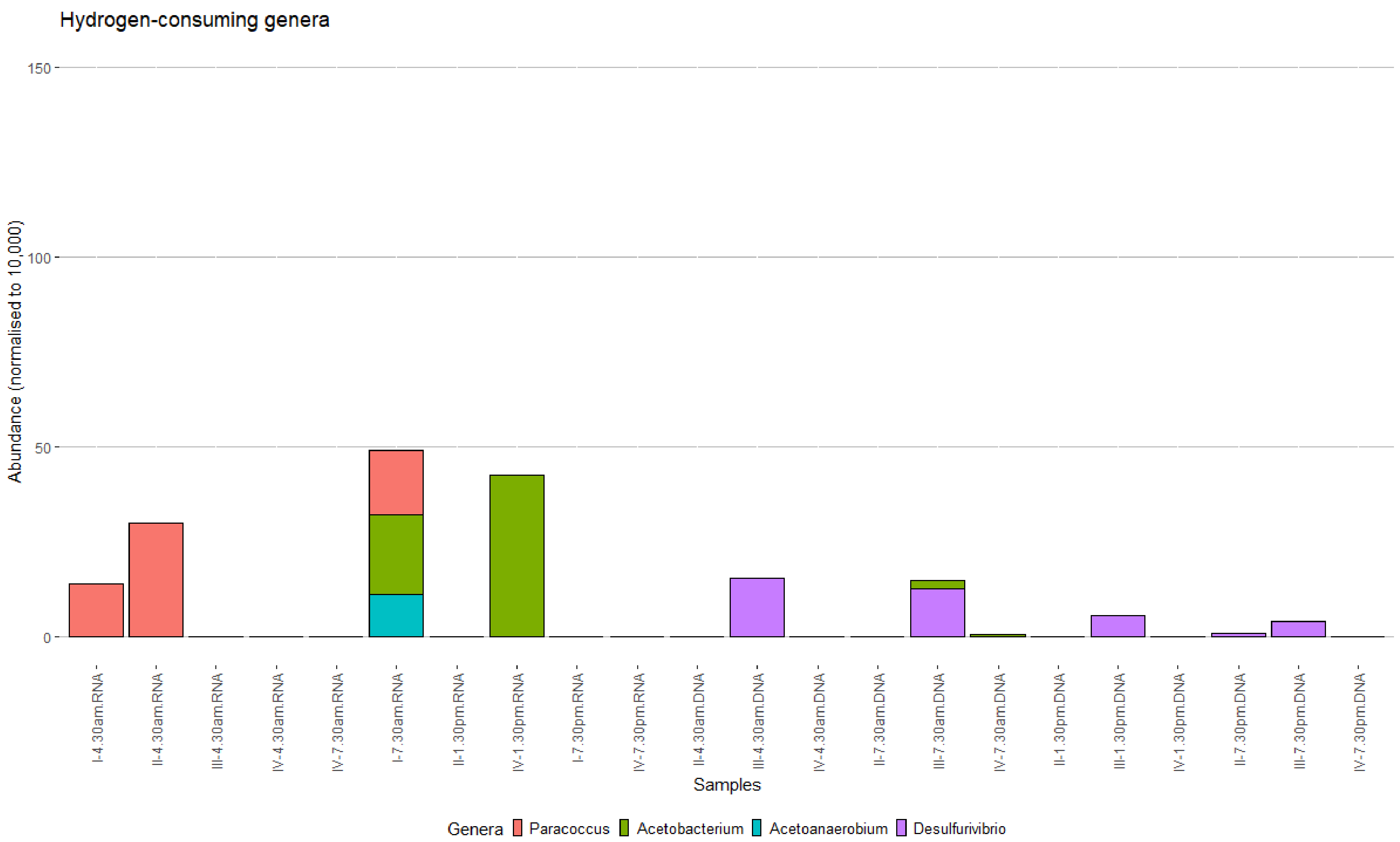
Hydrogen-consuming genera
Abundance (normalised to 10,000)
150
100
50
0
I-4.30am.RNA
II-4.30am.RNA
III-4.30am.RNA
IV-4.30am.RNA
IV-7.30am.RNA
I-7.30am.RNA
II-1.30pm.RNA
IV-1.30pm.RNA
I-7.30pm.RNA
IV-7.30pm.RNA
II-4.30am.DNA
III-4.30am.DNA
IV-4.30am.DNA
II-7.30am.DNA
III-7.30am.DNA
IV-7.30am.DNA
II-1.30pm.DNA
III-1.30pm.DNA
IV-1.30pm.DNA
II-7.30pm.DNA
III-7.30pm.DNA
IV-7.30pm.DNA
Samples
Genera
Paracoccus
Acetobacterium
Acetoanaerobium
Desulfurivibrio

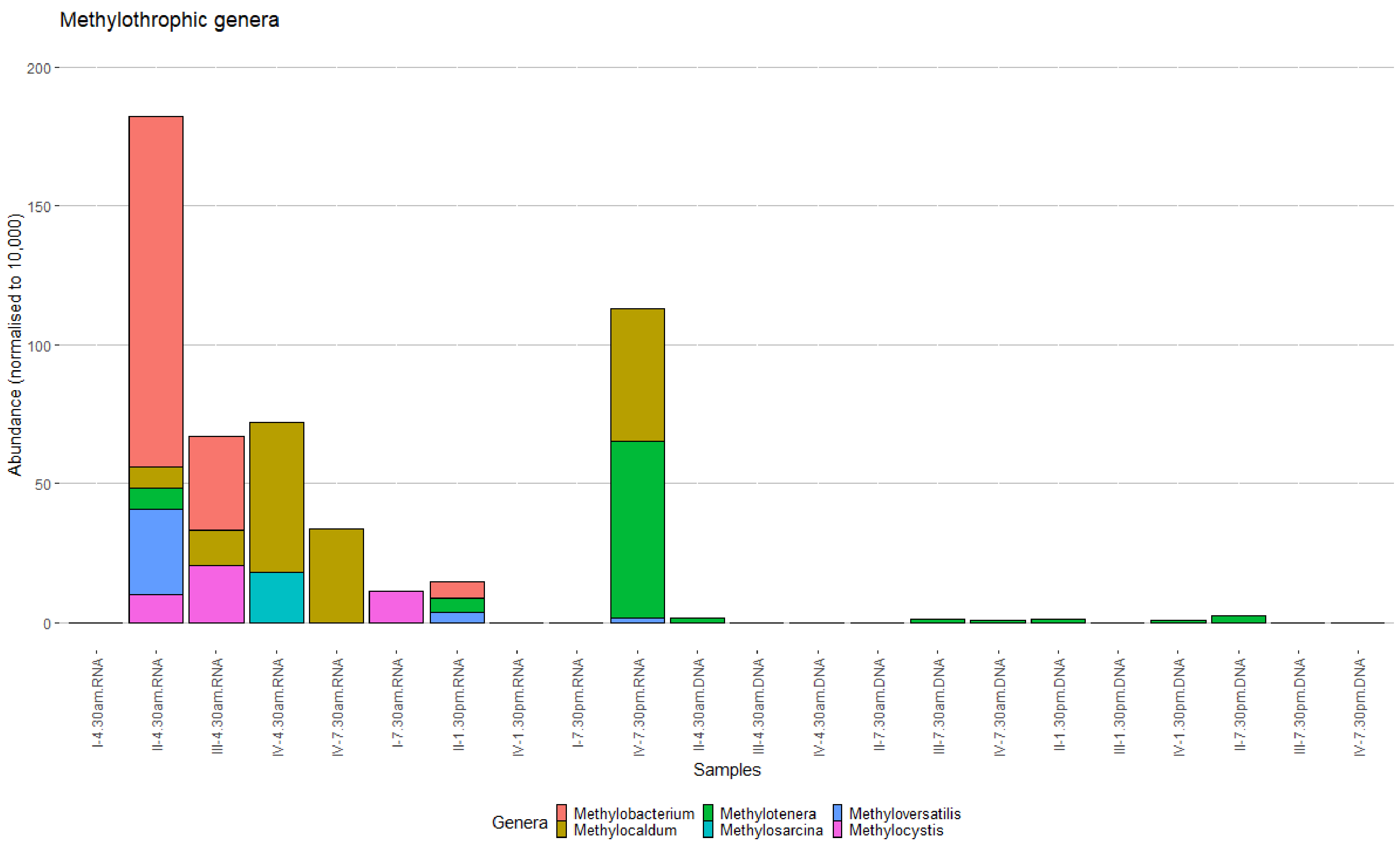
Methylothrophic genera
Abundance (normalised to 10,000)
200
150
100
50
0
I-4.30am.RNA
II-4.30am.RNA
III-4.30am.RNA
IV-4.30am.RNA
IV-7.30am.RNA
I-7.30am.RNA
II-1.30pm.RNA
IV-1.30pm.RNA
I-7.30pm.RNA
IV-7.30pm.RNA
II-4.30am.DNA
III-4.30am.DNA
IV-4.30am.DNA
II-7.30am.DNA
III-7.30am.DNA
IV-7.30am.DNA
II-1.30pm.DNA
III-1.30pm.DNA
IV-1.30pm.DNA
II-7.30pm.DNA
III-7.30pm.DNA
IV-7.30pm.DNA
Samples
Genera
Methylobacterium
Methylocaldum
Methylotenera
Methylosarcina
Methyloversatilis
Methylocystis

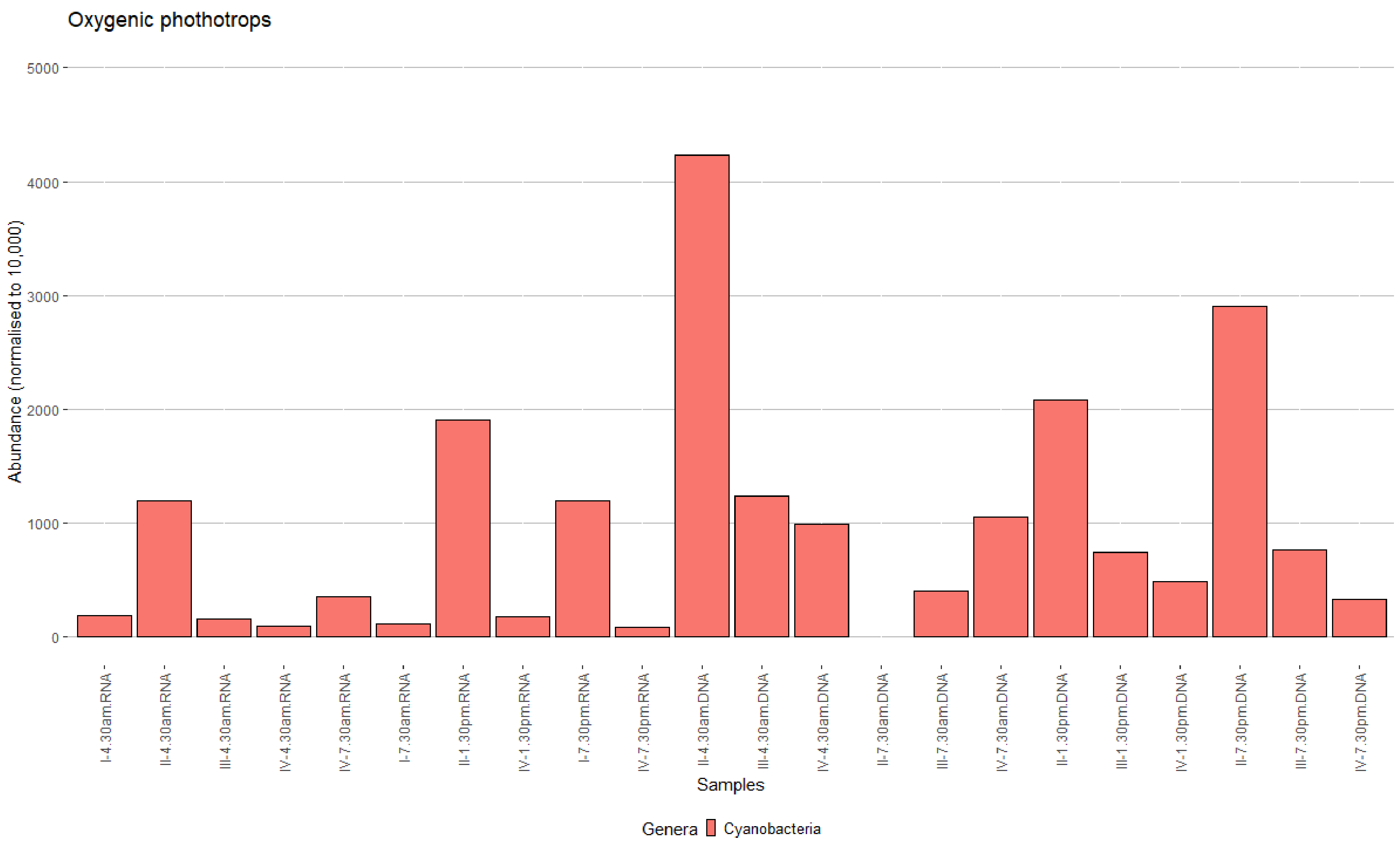
Oxygenic phothotrops
Abundance (normalised to 10,000)
5000
4000
3000
2000
1000
0
I-4.30am.RNA
II-4.30am.RNA
III-4.30am.RNA
IV-4.30am.RNA
IV-7.30am.RNA
I-7.30am.RNA
II-1.30pm.RNA
IV-1.30pm.RNA
I-7.30pm.RNA
IV-7.30pm.RNA
II-4.30am.DNA
III-4.30am.DNA
IV-4.30am.DNA
II-7.30am.DNA
III-7.30am.DNA
IV-7.30am.DNA
II-1.30pm.DNA
III-1.30pm.DNA
IV-1.30pm.DNA
II-7.30pm.DNA
III-7.30pm.DNA
IV-7.30pm.DNA
Samples
Genera
Cyanobacteria

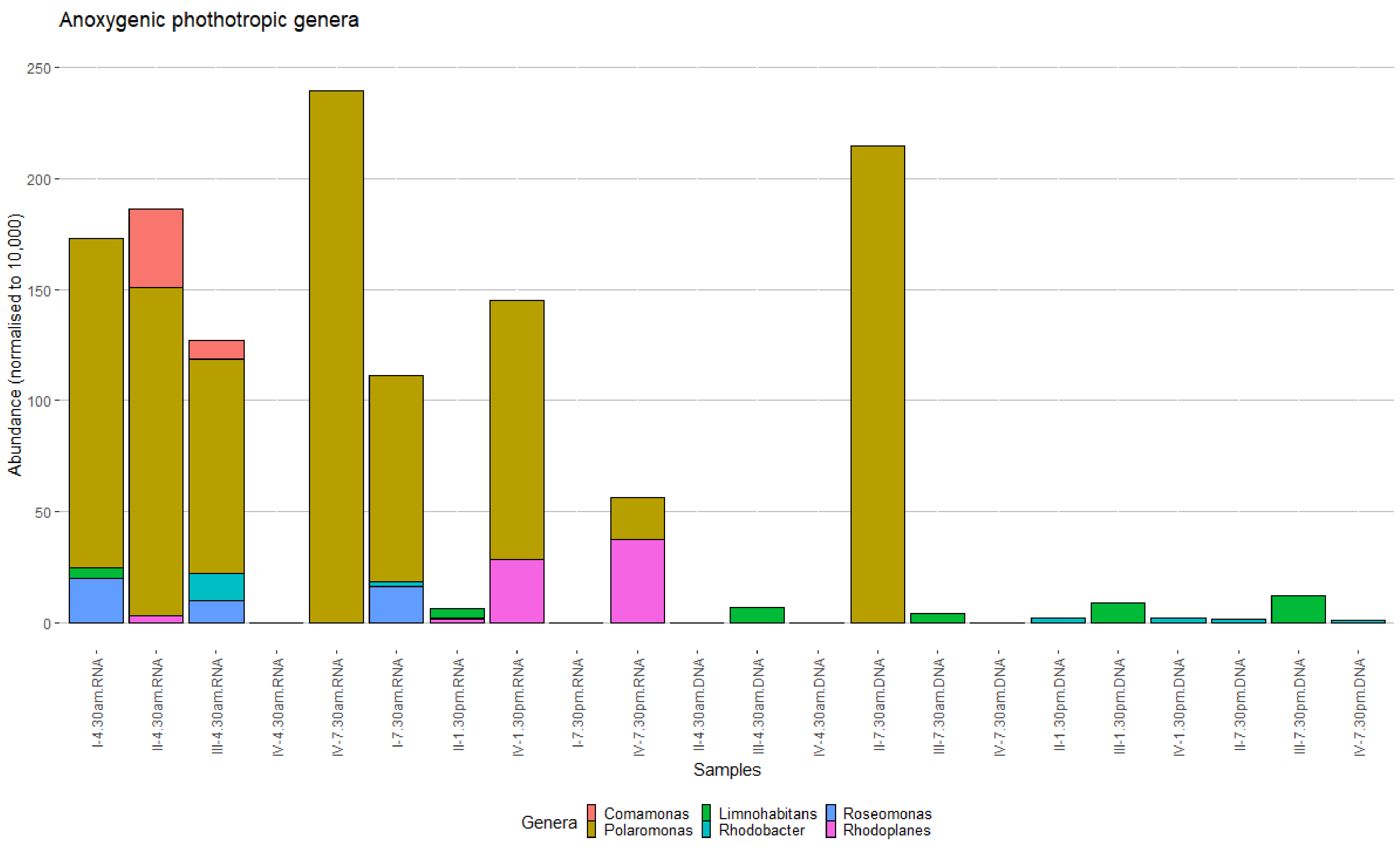
Anoxygenic phothotropic genera
Abundance (normalised to 10,000)
250
200
150
100
50
0
I-4.30am.RNA
II-4.30am.RNA
III-4.30am.RNA
IV-4.30am.RNA
IV-7.30am.RNA
I-7.30am.RNA
II-1.30pm.RNA
IV-1.30pm.RNA
I-7.30pm.RNA
IV-7.30pm.RNA
II-4.30am.DNA
III-4.30am.DNA
IV-4.30am.DNA
II-7.30am.DNA
III-7.30am.DNA
IV-7.30am.DNA
II-1.30pm.DNA
III-1.30pm.DNA
IV-1.30pm.DNA
II-7.30pm.DNA
III-7.30pm.DNA
IV-7.30pm.DNA
Samples
Genera
Comamonas
Polaromonas
Limnohabitans
Rhodobacter
Roseomonas
Rhodoplanes

Figure S2 - ***Barplot of the relative abundance of bacterial genera (Cyanobacteria are reported at class level) for the investigated metabolic traits. Genera whose abundance was <0.1% are not reported*.**

Table S1 – Copy gene/transcript abundance (standard error) per g of sediment of 16s RNA and *nar*G for samples III-7.30am, III-1.30pm, IV-7.30pm

Sample	16S rRNA genes	16S rRNA transcripts	*nar*G gene	*nar*G transcripts
**III-7.30am**	4.3 (2.5) x 10^{9}	1.3 (0.1) x 10^{6}	2.3 (1.0) x 10^{8}	ND
**III-1.30pm**	6.1 (1.3) x 10^{9}	1.6 (0.1) x 10^{6}	4.4 (1.7) x 10^{8}	1.1 (0.1) x 10^{9}
**IV-7.30pm**	5.9 (0.4) x 10^{10}	8.9 (0.6) x 10^{5}	2.5 (0.2) x 10^{9}	ND

ND: not detected
